# Phrenic stimulation decreases brain injury biomarkers in sedated mechanically ventilated patients: preliminary observations

**DOI:** 10.1186/s13054-025-05435-2

**Published:** 2025-05-26

**Authors:** Bassi Thiago, Rohrs Elizabeth, Parfait Melodie, Hannigan Brett, Reynolds Steve, Mayaux Julien, Decavèle Maxens, Demoule Alexandre, Similowski Thomas, Dres Martin

**Affiliations:** 1 Interdepartmental Division of Critical Care Medicine, University of Toronto, Toronto, Canada; 2 Lungpacer Medical Inc., Vancouver, Canada; 3 Biomedical, Physiology and Kinesiology, Simon Fraser University, Burnaby, Canada; 4 Sorbonne Université, INSERM, UMRS1158 Neurophysiologie respiratoire expérimentale et clinique, Paris, France; 5 AP-HP. Sorbonne Université, Hôpital Pitié-Salpêtrière–Service de Médecine Intensive et Réanimation, 75013 Paris, France; 6 Department of Health Sciences and Technology, ETH Zurich, Zurich, Switzerland; 7 AP-HP. Sorbonne Université, Hôpital Pitié-Salpêtrière–(Département R3S), 75013 Paris, France

**Keywords:** Biomarkers, Phrenic nerve stimulation, Neuromodulation, ARDS, Astrocytes

## Brief abstract

Astrocytes assist in modulating the breathing cycle. Mechanically ventilated sedated patients have their respiratory drive suppressed, which may affect astrocytes’ function. This pilot study showed that phrenic nerve stimulation may protect astrocyte function and activity, maintaining the integrity of the blood–brain barrier in these patients. 

Neural control of respiratory rhythm is a complicated process controlled by both neuron and glia cells across subcortical and cortical networks, sending rhythmic or non-rhythmic inputs to respiratory motoneurons in the spinal cord [1]. Glial cells, specifically astrocytes, also play a major role in controlling blood–brain barrier permeability, acting as “the brain’s gatekeepers” [1]. Cervical vagus nerve stimulation and the use of mechanical ventilation have been shown to modify the blood–brain barrier permeability [2, 3]. Better ventilation distribution and increased alveolar ventilation secondary to diaphragm activity in mechanically ventilated patients could enrich the afferent traffic from the respiratory system to the brain through pulmonary stretch receptors stimulation. This could modulate the activity of the tractus solitarius nucleus directly via the vagal pathway or indirectly via spinal-to-brainstem communication. Phrenic stimulation could thus upregulate astrocyte function and subsequently affect the permeability and integrity of the blood–brain barrier. This dynamic interaction is essential for normal brain function and plays a significant role in neurological health and disease states, which can be affected by deep sedation.

Deep sedation is one strategy employed in the delivery of invasive mechanical ventilation to ensure patient-ventilator synchrony. Although mechanical ventilation saves lives, it is associated with negative effects on pulmonary air distribution, diaphragm function, cardiac function and an increased likelihood of delirium and cognitive impairment [4, 5]. Increased levels of biomarkers of astrocytes and neuronal injuries have been associated with cognitive impairment and delirium [6]. Calcium binding S100β and glial fibrillary acid protein (GFAP) are examples of astrocyte biomarkers while light chain neurofilament (NfL), tau protein (tau), neuro specific enolase (NSE), and ubiquitin c-terminal hydrolase L-1 (UCHL-1) are neuronal biomarkers [1, 6]. Recently, biomarkers for brain injury have been used to quantify astrocytes and neuronal injury in traumatic brain injured patients. The American Food and Drugs Administration approved the use of biomarkers for astrocytes and neuronal injury to assist in recognizing mild brain injuries (Glasgow coma scale between 14 and 15) that need imagining investigation. Also, S100β, GFAP, NSE and UCHL-1 biomarkers have been used to follow up with the resolution of concussion in traumatic brain injury patients who scored 15 on the Glasgow coma scale [7, 8].

In this research letter, we report the effects of continuous bilateral phrenic nerve stimulation synchronized with the inspiratory phase of the respiratory cycle on biomarkers for brain injury in twelve critically ill acute respiratory distress syndrome (ARDS) deeply sedated (not paralyzed) mechanically ventilated patients. The data was derived from a crossover hypothesis-generating study approved by the ethics Comité de Protection de Personnes, Ile-De-France, Paris, France on June 15th, 2021 (ClinTrials.gov: NCT04844892). Phrenic nerve stimulation was achieved by a central-line catheter embedded with electrodes (Lungpacer Medical Inc., Canada) inserted into the left subclavian or left internal jugular veins. The study protocol consisted of four 60-min sessions, with sessions 1 & 3 unpaced and 2 & 4 paced [9]. All patients were studied during the daytime to avoid circadian cycle interference in the results. Following the previous method reported, stimulation was delivered as trains of pulseswith a pulse frequency of 40 Hz and a pulse width between 200 and 300 microseconds [10]. Stimulation trains were set to be 100 microseconds shorter than the inspiratory time set on the ventilator [10]. For instance, if the inspiratory time set on the ventilator was 0.9 s, the stimulation train was set to 0.8 s to avoid dysynchrony between diaphragm contraction and inspiratory flow [10]. The maximum total current delivered could not exceed 27 mA [10]. Multipolar electrodes were used to map the electrodes with the lowest and highest stimulation threshold, then bipolar electrodes were set to deliver phrenic nerve stimulation to achieve a time-product reduction between 10 and 20%. [10] All patients were ventilated in volume control, with the ventilatory settings kept constant during the study. Sedation infusions were also kept unchanged during the sessions (Table 1). Blood samples for brain biomarkers (S100β, GFAP, UCHL-1, NSE, NfL and tau) were collected at baseline and the end of each session. We conducted an analysis comparing serum concentration between the sessions using a paired Friedman test. Data from this trial has been previously published elsewhere, showing greater dorsal pulmonary air distribution, greater cardiac index and greater brain activity and connectivity during the phrenic nerve stimulation sessions compared to control sessions [9, 10]. Two biomarkers analyzed showed statistical significance. Serum concentrations of biomarkers of brain injury reported as median (interquartile range) from study sessions 1 to 4 (Table 2). GFAP and S100β serum concentrations were statistically significantly reduced during the paced sessions when compared to unpaced sessions (Fig. 1). There was no significant change in the serum concentration of biomarkers for neuronal injury from sessions 1 to 4. In a previous study, we reported that phrenic stimulation in synchrony with invasive mechanical ventilation in deeply sedated, moderate ARDS patients increased alpha and gamma frequencies cortical activity, brain connectivity, and neuronal synchronization in the frontal–temporal-parietal cortices compared to non-phrenic stimulation sessions [10]. The increase in alpha and gamma frequencies and the activation of the frontal–temporal-parietal cortices resemble those observed in studies on diaphragmatic breathing in awake, healthy participants [10]. These results may indicate that restoring phrenic nerve activity in deeply sedated mechanically ventilated patients may protect astrocyte function and activity, maintaining the integrity of the blood–brain barrier. Preclinical studies have shown that 50 h of phrenic nerve stimulation in deeply sedated mechanically ventilated pigs mitigates astrocytes and microglia activation preventing hippocampal cellular apoptosis and leading to lower serum concentrations of biomarkers for astrocyte injury (S100β and GFAP) and neuronal injury (UCHL-1) compared to pigs receiving mechanical ventilation only (i.e., control) [11]. Studies showed that patients with delirium have greater serum concentrations of astrocytes (S100β and GFAP) and neuronal injury (tau and NfL) biomarkers compared to patients without delirium [6]. Although GFAP greater than 250 pg/ml have been associated with positive CT scans (i.e., the presence of cerebral contusion) in the traumatic brain injury population, it is unclear whether a reduction observed in the GFAP values here reported may result in clinical benefits [12]. Also, the absence of significant changes in the biomarkers for neuronal injury may be due to a longer half-life compared to astrocyte markers. Therefore, four hours may not have been enough to observe any potential changes in these biomarkers. While promising and preliminary in nature, the results presented here may indicate the importance of either phrenic nerve stimulation or spontaneous breathing in brain protection in critically ill patients. The coherence between the previously reported EEG data [10] and the current biological data provides a strong incentive and encouragement to conduct larger-scale human studies that would first corroborate the effects of phrenic stimulation on the brain and then try to relate these effects with clinical outcomes such as delirium and cognitive dysfunction. Whether such putative effects depend on phrenic stimulation or can result in the preservation of a brain-lung dialogue by spontaneous breathing will be the next highly clinically relevant question.

**Table 1:** Patients’ characteristics

Characteristics	N = 12
Age (years)	57 (47–70)
Male sex, n (%)	9 (69)
Body mass index, kg/m^2^	26 (20–31)
Simplified acute physiology score 2	45 (38–52)
Norepinephrine at inclusion, μg kg^–1^ min^–1^	0.10 (0.04–0.17)
Richmond agitation sedation scale	− 4 (− 4 to − 5)
Comorbidities	n (%)
Chronic pulmonary disease	2 (20)
Active immunosuppression	4 (40)
Risk factor of ARDS	n (%)
SARS-CoV-2	4 (31)
Aspiration pneumonia	4 (31)
Community acquired pneumonia	3 (24)
Tuberculosis	1 (8)
Acute pancreatitis	1 (8)
*Respiratory mechanics*	
Tidal volume, ml/kg of predicted body weight	5.9 (5.7–6.1)
Respiratory rate, min^–1^	26 (24–30)
Positive end expiratory pressure, cm H_2_O	8 (8–10)
Respiratory system driving pressure, cm H_2_O	16 (14–17)
Respiratory system compliance, ml/cm H_2_O	26 (22–33)
Lung compliance, cm H_2_O	37 (30–43)
*Arterial blood gas*	
Lactate, mmol/l	1.0 (0.9–1.7)
PaCO_2_, mmHg	42 (38–47)
PaO_2_/FiO_2_	163 (144–187)

Data are presented as n (%) and median (25–75 interquartile). Respiratory mechanics were obtained before starting the protocol, and an average of three measurements was provided for driving pressure, respiratory system compliance, and lung compliance. ARDS, acute respiratory distress syndrome; FiO_2_, the fraction of inspired oxygen

**Table 2:** Serum concentrations of biomarkers of brain injury are reported as median (interquartile range) from study sessions 1–4. Sessions 1 and 3 are unpaced, and sessions 2 and 4 are paced

	Session 1	Session 2	Session 3	Session 4
S100β	0.11 pg/ml (0.09–0.14)	0.08 pg/ml (0.07–0.14)	0.09 pg/ml (0.07–0.11)	0.07 pg/ml (0.06–0.11)
GFAP	169 ng/ml (103–552)	114 ng/ml (80–486)	202 ng/ml (80–571)	130 ng/ml (78–272)
UCHL-1	45 ng/ml (37–114)	40 ng/ml (28–132)	51 ng/ml (21–193)	44 ng/ml (33–190)
NSE	14 pg/ml (7.8–16)	11 pg/ml (8.3–16)	12 pg/ml (8–16)	11 pg/ml (8–15)
NfL	95 pg/ml (74–235)	109 pg/ml (70–227)	98 pg/ml (71–209)	80 pg/ml (56–240)
tau	2.0 pg/ml (1.0–2.6)	1.2 pg/ml (0.8–2.2)	1.2 pg/ml (0.6–1.6)	2.0 pg/ml (0.8–2.6)

**Fig. 1:**
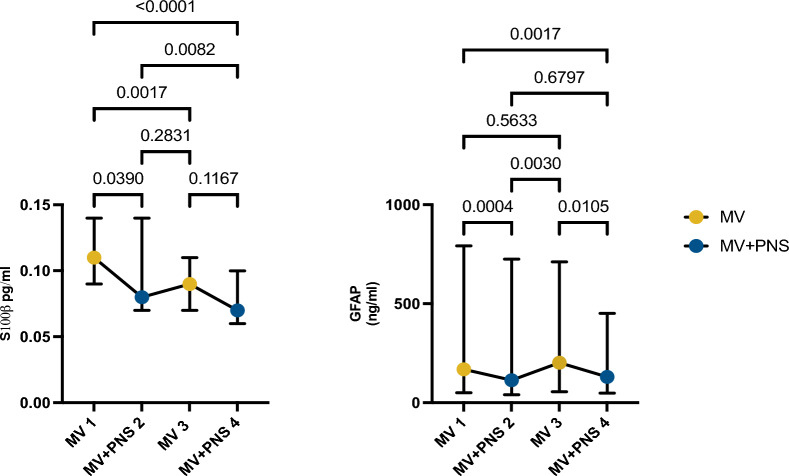
Dot plots reported as median and interquartile range showing the serum concentration of biomarkers for astrocyte injury from sessions 1–4. Calcium binding S100β serum concentration and Glial fibrillary acid protein (GFAP) serum concentration were reported from sessions 1–4

## Data Availability

The datasets generated during and/or analysed during the current study are available from the corresponding author upon reasonable request.
